# Radioembolization versus portal vein embolization for contralateral liver lobe hypertrophy: effect of cirrhosis

**DOI:** 10.1007/s00261-021-03048-1

**Published:** 2021-03-29

**Authors:** Heiner Nebelung, Thomas Wolf, Sebastian Bund, Christoph Georg Radosa, Verena Plodeck, Sabine Grosche-Schlee, Carina Riediger, Ralf-Thorsten Hoffmann, Jens-Peter Kühn

**Affiliations:** 1 Department of Radiology, Dresden University Hospital, Fetscherstr. 74, 01307 Dresden, Germany; 2 Department of Nuclear Medicine, Dresden University Hospital, Fetscherstr. 74, 01307 Dresden, Germany; 3 Department of Visceral, Thoracic and Vascular Surgery, Dresden University Hospital, Fetscherstr. 74, 01307 Dresden, Germany

**Keywords:** Therapeutic embolization, Hypertrophy, Liver cirrhosis, Portal vein, Hepatic artery

## Abstract

**Purpose:**

Preoperative hypertrophy induction of future liver remnant (FLR) reduces the risk of postoperative liver insufficiency after partial hepatectomy. One of the most commonly used methods to induce hypertrophy of FLR is portal vein embolization (PVE). Recent studies have shown that transarterial radioembolization (TARE) also induces hypertrophy of the contralateral liver lobe. The aim of our study was to evaluate contralateral hypertrophy after TARE versus after PVE taking into account the effect of cirrhosis.

**Methods:**

Forty-nine patients undergoing PVE before hemihepatectomy and 24 patients with TARE as palliative treatment for liver malignancy were retrospectively included. Semi-automated volumetry of the FLR/contralateral liver lobe before and after intervention (20 to 65 days) was performed on CT or MRI, and the relative increase in volume was calculated. Cirrhosis was evaluated independently by two radiologists on CT/MRI, and interrater reliability was calculated.

**Results:**

Hypertrophy after PVE was significantly more pronounced than after TARE (25.3% vs. 7.4%; *p* < 0.001). In the subgroup of patients without cirrhosis, the difference was also statistically significant (25.9% vs. 8.6%; *p* = 0.002), whereas in patients with cirrhosis, the difference was not statistically significant (18.2% vs. 7.4%; *p* = 0.212). After PVE, hypertrophy in patients without cirrhosis was more pronounced than in patients with cirrhosis (25.9% vs. 18.2%; *p* = 0.203), while after TARE, hypertrophy was comparable in patients with and without cirrhosis (7.4% vs. 8.6%; *p* = 0.928).

**Conclusion:**

TARE induces less pronounced hypertrophy of the FLR compared to PVE. Cirrhosis seems to be less of a limiting factor for hypertrophy after TARE, compared to PVE.

**Graphic abstract:**

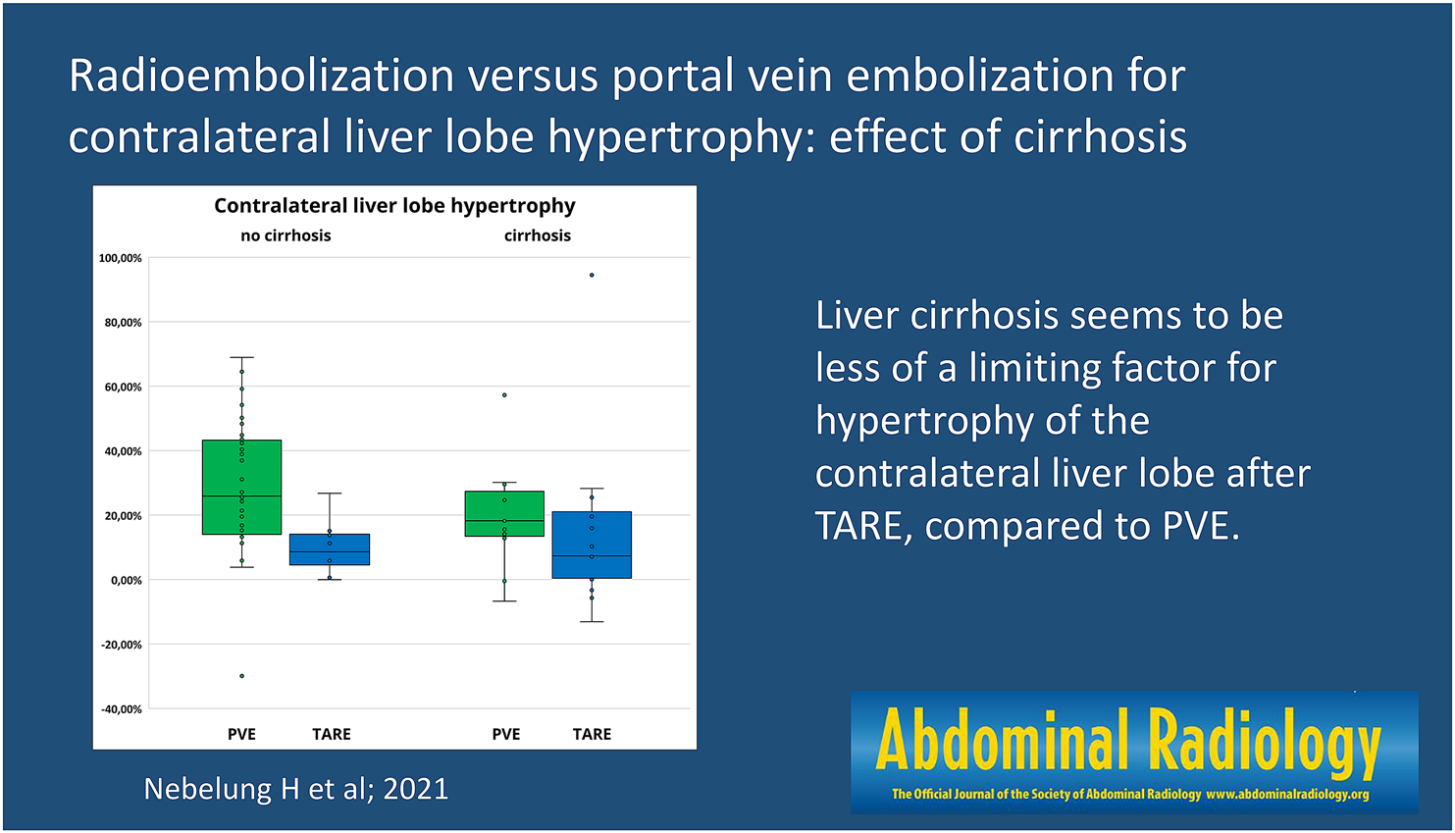

## Introduction

Partial hepatectomy is frequently the curative treatment of choice for malignant tumors of the liver. Depending on tumor size, location, and number of lesions, extensive resections up to extended hemihepatectomy can be necessary. Resection of more than 75% of hepatic parenchyma more than triples the risk of postoperative hepatic dysfunction [1]. Preoperative hypertrophy induction of the future liver remnant (FLR) can lead to an increase in functioning liver parenchyma and, therefore, reduces the risk of postoperative liver insufficiency. The currently best-studied methods to induce hypertrophy of FLR are portal vein embolization (PVE) and portal vein ligation [2, 3], which is often combined with partition of liver parenchyma (“in situ split”) [4]. Recent studies have shown that transarterial radioembolization (TARE) also induces hypertrophy of the contralateral, untreated liver lobe [5–7].

Physiologically, total hepatic blood flow consists of 70–80% portal venous and 20–30% hepatic arterial blood. In patients with cirrhosis, portal blood flow decreases and the “hepatic arterial buffer response” keeps total hepatic flow at a constant level by increasing hepatic arterial blood flow [8].

Occlusion of portal veins of the liver segments that will be resected leads to redirection of portal blood to the FLR. This way, PVE leads to hypertrophy of the untreated liver segments [9]. In general, there is high variability in hypertrophy after PVE. Many influencing factors have been discussed in the literature, e.g., intrahepatic tumor burden [10], hyperbilirubinemia [11], and BMI [12], but overall the physiology of liver hypertrophy is not well understood. In patients with cirrhosis, sufficient hypertrophy seems to take longer than in patients without cirrhosis [13]. A disadvantage of PVE is possible tumor progress during hypertrophy induction, since malignant tumors recruit their blood supply from arterial vessels [14].

TARE is currently used as a palliative treatment for malignant liver diseases. It allows for selective radiation therapy of hypervascular lesions [15]. As a result, local tumor control and downsizing can be achieved, possibly leading to resectability of the lesions. Several studies have shown that PVE induces more pronounced contralateral hypertrophy [6] and TARE-induced hypertrophy of the untreated liver lobe seems to take longer than after PVE [5], but there is little data on the effect of cirrhosis on hypertrophy after TARE. One study by Teo et al. showed a significantly greater degree of hypertrophy after TARE in HCC patients with hepatitis B, compared to those with hepatitis C or alcoholic cirrhosis [7]. Another study by Edeline et al. stated that TARE induces similar increases in FLR volume compared to PVE in patients with cirrhosis [16]. A recent review by Birgin et al. analyzed a total of 16 studies, comprising 602 patients, and stated that the influence of cirrhosis on hypertrophy of the contralateral liver lobe after TARE remains controversial, with no significant difference of kinetic growth rate in the three included studies which assessed only patients with cirrhosis, compared to patients without cirrhosis [17].

Thus, the aim of our study was to evaluate the effect of cirrhosis on contralateral liver lobe hypertrophy after TARE versus after PVE.

## Materials and methods

### Study population and indications

**Fig. 1:**
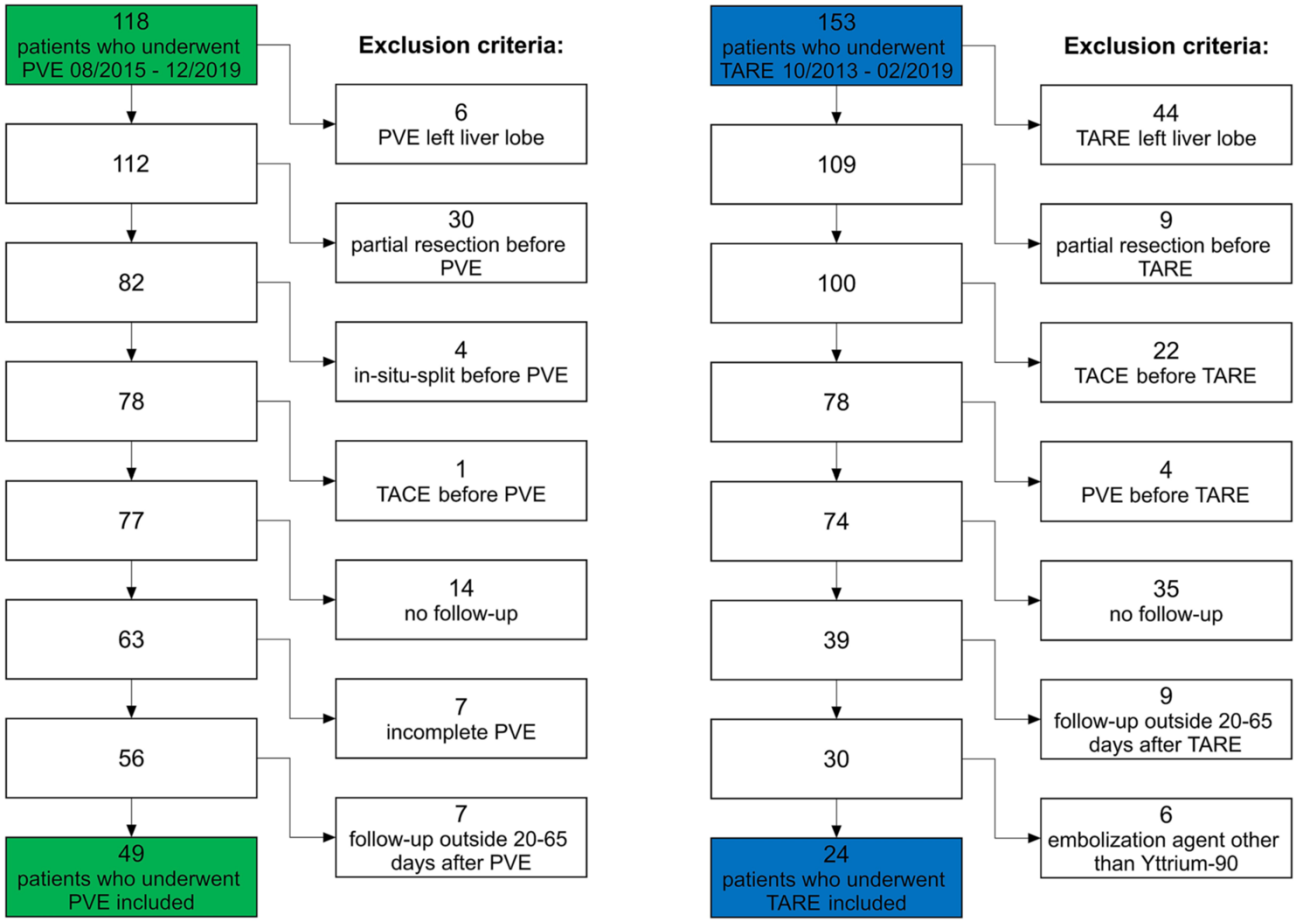
Study population. After applying exclusion criteria, 49 of 118 patients who underwent PVE from 08/2015 to 12/2019 and 24 of 153 patients who underwent TARE from 10/2013 to 02/2019 were included

This study was approved by the local ethics committee. Due to the retrospective evaluation, written informed consent was waived. The radiology information system of our hospital was searched for patients who underwent PVE before hemihepatectomy in curative intention between August 2015 and December 2019 and patients who underwent TARE as a palliative treatment for malignant liver diseases between October 2013 and February 2019. Inclusion criteria were intervention of the right liver lobe (± segment IV), preinterventional CT or MRI, and postinterventional CT or MRI between 20 and 65 days after TARE or PVE. Postinterventional imaging was performed prior to surgery after PVE and along in-hospital follow-up protocols after TARE. Exclusion criteria were preinterventional partial liver resections, preinterventional in situ split, a prior transarterial chemoembolization or prior PVE. Some patients were excluded because they underwent resection or second interventional treatment without intermediary follow-up. Further exclusion criteria were incomplete PVE in the PVE group and treatment with embolization agents other than Yttrium-90 in the TARE group. Seventy-three patients fulfilled all inclusion criteria, including 49 patients who underwent PVE and 24 patients who underwent TARE (Fig. 1; Table 1).

**Table 1:** Baseline characteristics

Baseline characteristics		PVE	TARE
		n = 49	n = 24
Sex	m	34 (69%)	20 (83%)
	f	15 (31%)	4 (17%)
Age (median (IQR))		66 y (IQR 13 y)	71.5 y (IQR 13.25 y)
Indication	Hepatocellular carcinoma	13 (27%)	15 (63%)
	Cholangiocellular carcinoma	24 (49%)	2 (8%)
	Metastases	12 (24%)	7 (29%)
Child–Pugh-Score	A	9 (82%)	12 (75%)
	B	2 (18%)	4 (25%)
	C	0 (0%)	0 (0%)
		(n= 11*)	(n = 16*)

^*^Child-Pugh score was evaluated only in patients who were assessed as having cirrhosis (PVE: 11 out of 49; TARE: 16 out of 24)

### Technique of interventions

All interventions were performed on an image-guided therapy system (Philips Azurion 7 M20, Philips Healthcare).

*PVE* After ultrasound-guided (Philips Affiniti 70G, Philips Healthcare) puncture (chiba needle, Peter Pflugbeil GmbH) of a right portal vein branch, a 5F sheath (DilPlus, Peter Pflugbeil GmbH) is introduced and the main portal vein accessed via a guidewire (Nitrex Guidewire, Medtronic). Using a Simmons catheter (SIM-catheter, Cardinal Health), a diagnostic portogram is performed to assess portal vein anatomy. After placing the Simmons catheter in the right portal vein, a microcatheter (Progreat, Terumo GmbH) is then placed in the anterior and posterior pedicle of the right portal vein (in some cases also the portal vein branch supplying segment IV) and embolization is performed using a 1:5 mixture of histoacryl (Histoacryl®, B. Braun Melsungen AG) and lipiodol (Lipiodol® Ultra-Fluid, Guerbet GmbH).

*TARE* After puncture (puncture needle, Cardinal Health) of the common femoral artery, introduction of a 5F sheath (Glidesheath Slender, Terumo Deutschland GmbH), and cannulation of the celiac trunk (Cobra-catheter, Cardinal Health), diagnostic angiography is performed. Collaterals, which might lead to unwanted spread of the radioembolization agent into other organs (mainly via the gastroduodenal artery), are identified and occluded using coil embolization where appropriate. The right hepatic artery is cannulated using a microcatheter (Progreat, Terumo GmbH). Technetium-99m-HSA microspheres (ROTOP HSA Mikrosphären B20, ROTOP) are applied to assess the distribution. In a second session, Yttrium-90 microspheres (SIR-Spheres Resin Microspheres, Sirtex Medical) are applied selectively into the right hepatic artery. To estimate each individual dosage of Yttrium-90 microspheres for SIRT, several technical and clinical considerations are taken into account, as described previously by Salem et al. [18]. Using pretreatment diagnostic CT or MR imaging, diagnostic angiography and Technetium-99m-MAA SPECT/CT scanning, activities are calculated according to the body surface area method and/or the partition model, if applicable [19]. Thus, therapy dosages are adjusted in respect to the tumor mass, the uninvolved liver parenchyma, the lung shunt fraction, and the patient’s clinical status, respectively. Planned dosages are reduced up to 20% in patients with pre-existing liver damage to prevent radioembolization-induced liver disease [20]. In our study, median administered activity for patients undergoing TARE was 1100 MBq (IQR 1670 MBq).

### Evaluation of cirrhosis

Cirrhosis (present/not present) was evaluated independently by two radiologists on CT/MRI (one radiologist with more than 15 years of experience in liver imaging, one resident with 2 years of experience in liver imaging). They were blinded to the type of intervention (TARE or PVE). They used the following criteria: hypertrophy of the caudate lobe and segments II/III with concomitant atrophy of segments VI/VII [21], surface and parenchymal nodularity and heterogeneity, portal vein enlargement, portal venous thrombosis, and ascites [22]. In case of disagreement, the result was decided in consensus.

### Volumetry

**Fig. 2:**
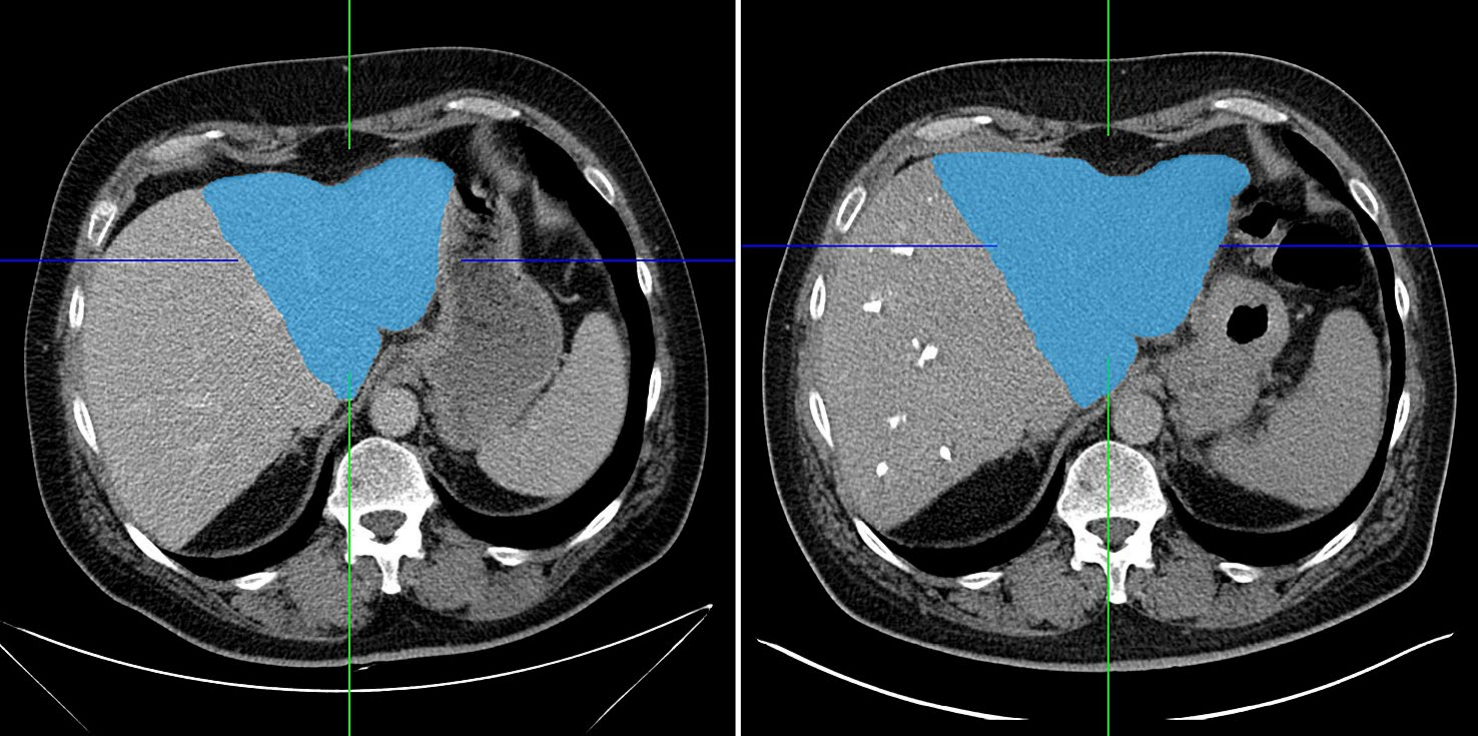
CT scans before (left) and about two months after PVE (right) with volumetry of the FLR (blue)

**Fig. 3:**
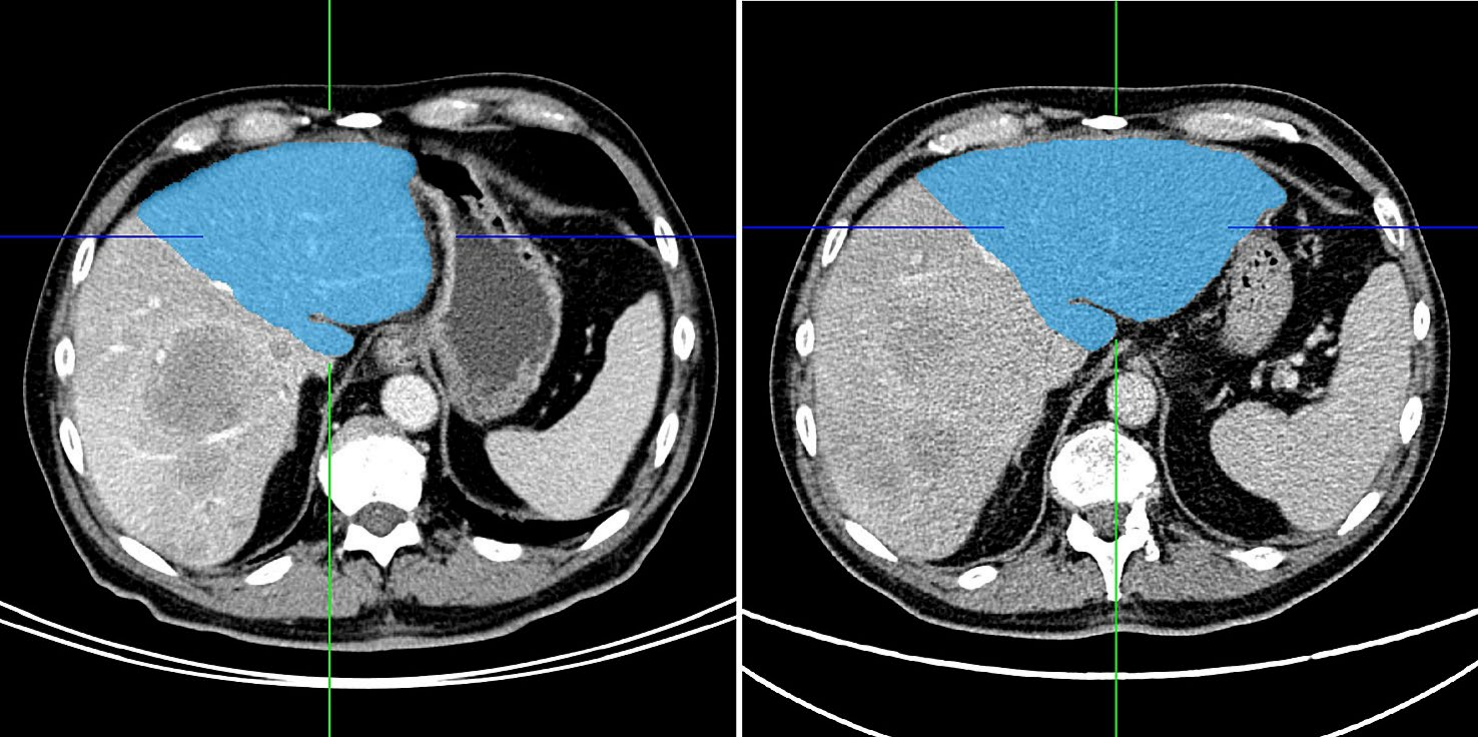
CT scans before (left) and about two months after TARE (right) with volumetry of the contralateral liver lobe (blue)

Volumetry of the FLR before and after PVE or the contralateral liver lobe before and after TARE was performed using the contour segmentation tool with semi-automated edge detection of IMPAX (IMPAX EE R 20, Agfa HealthCare) by two independent raters. Both were trained prior to the evaluation by a radiologist with more than 15 years of experience in liver imaging (Figs. 2, 3).

### Statistics

At first, all variables to be compared were examined for normal distribution by the Shapiro–Wilk test [23]. Since in most cases there was no normal distribution, medians and interquartile ranges (IQR) were calculated and non-parametric tests were used for all comparisons [24].

Relative increase in volume was calculated and compared using the Mann–Whitney *U* test. The significance level was set at *α* = 0.05 in all cases. Time interval between intervention and postinterventional CT/MRI was documented.

Regarding the evaluation of cirrhosis, interrater reliability was evaluated using Cohens kappa (κ) [25, 26], which was interpreted according to the recommendations of Landis and Koch [27] (κ ≤ 0 poor; 0.01–0.20 slight, 0.21–0.40 fair, 0.41–0.60 moderate, 0.61–0.80 substantial and 0.81–1.00 almost perfect agreement).

The Shapiro–Wilk test, Mann–Whitney *U* test, and the calculation of Cohens kappa were performed using SPSS (IBM Corp. Released 2018. IBM SPSS Statistics for Windows, Version 26.0). Diagrams and tables were created using Microsoft Office Excel 2016 (Microsoft Corporation).

## Results

Out of 49 patients who underwent PVE, 11 patients were assessed as having cirrhosis (22%), 38 patients as not having cirrhosis (78%). Out of 24 patients who underwent TARE, there were 16 patients with cirrhosis (67%) and 8 patients without (33%).

**Fig. 4:**
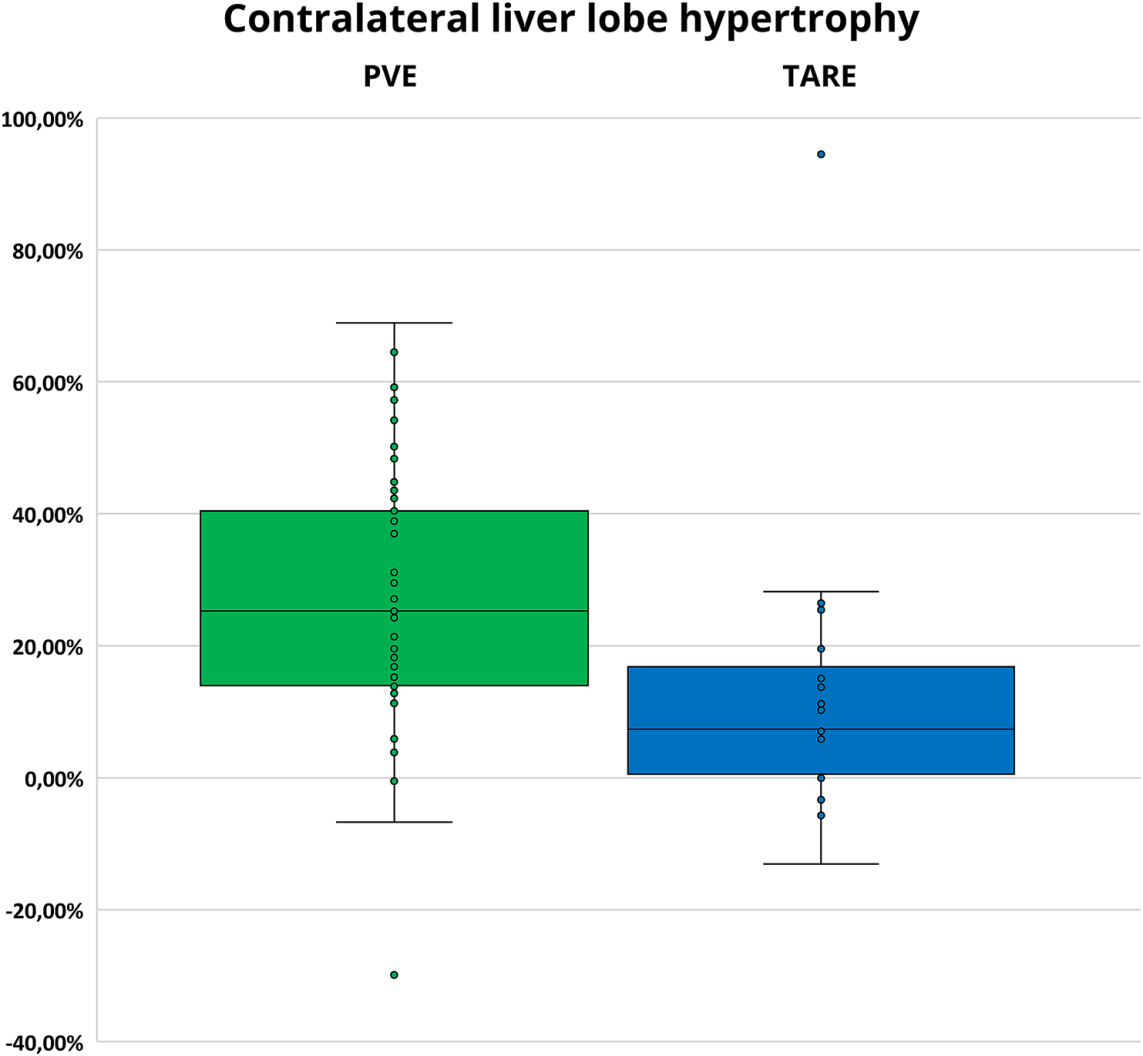
Contralateral liver lobe hypertrophy in patients who underwent PVE and in patients who underwent TARE

In general, hypertrophy after PVE was significantly more pronounced than after TARE (25.3% (IQR 27.4%) vs. 7.4% (IQR 18.1%); *p* < 0.001), although time intervals between PVE and postinterventional CT/MRI were shorter than between TARE and postinterventional CT/MRI [33 days (IQR 16 days) vs. 52.5 days (IQR 16 days)] (Fig. 4; Table 2).

**Table 2:** Contralateral liver lobe hypertrophy in patients who underwent PVE and in patients who underwent TARE

	Contralateral liver lobe hypertrophy Median (Interquartile range)	
	Hypertrophy	Time interval
PVE	25.3% (27.4%)	33 days (16 days)
TARE	7.4% (18.1%)	52,5 days (16 days)
	*p* < 0.001	

**Fig. 5:**
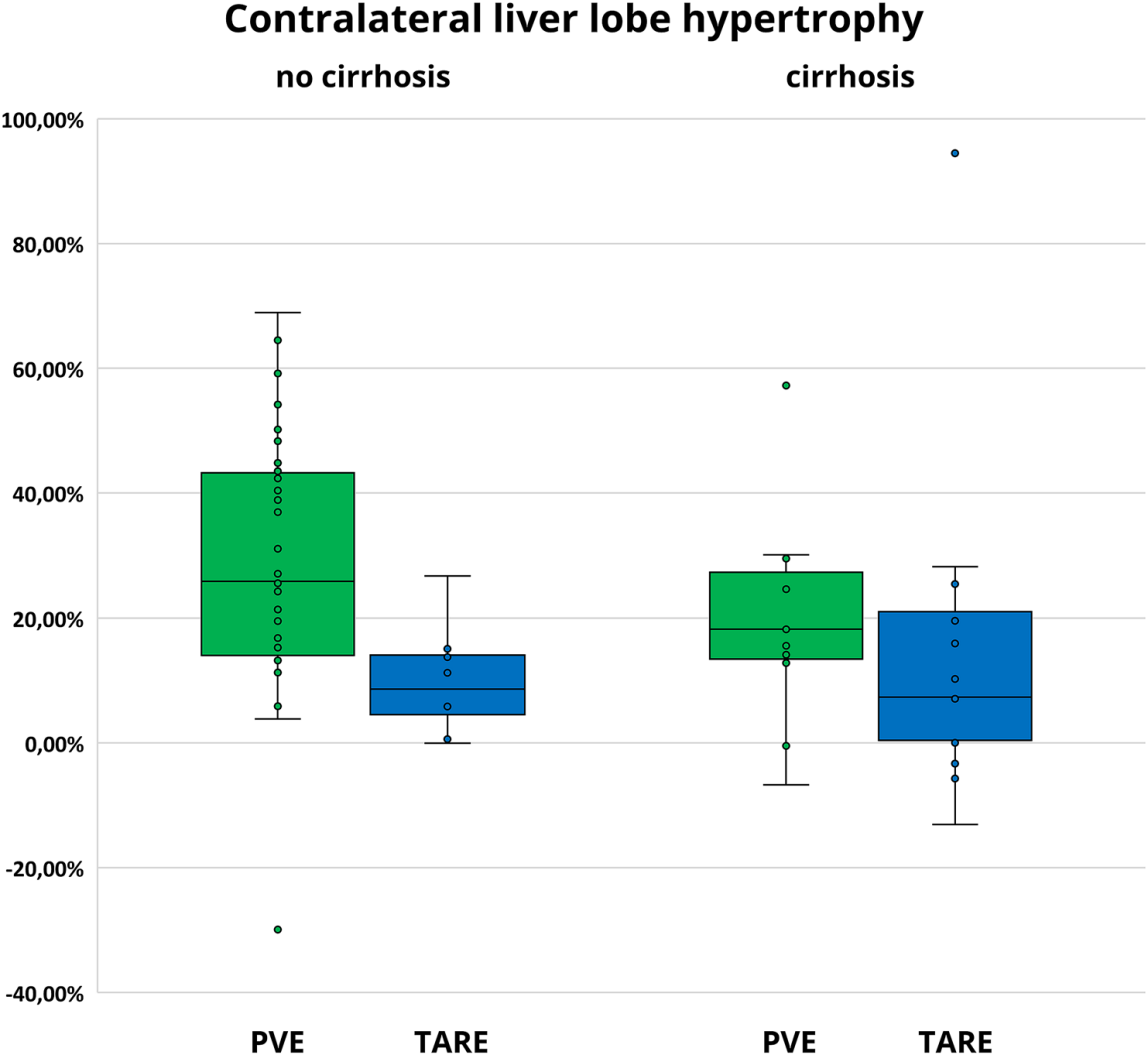
Contralateral liver lobe hypertrophy in patients with cirrhosis and without (after PVE and after TARE)

In the subgroup of patients without cirrhosis, hypertrophy after PVE was significantly more pronounced than after TARE [25.9% (IQR 29.8%) vs. 8.6% (IQR 12.8%); *p* = 0.002], whereas time intervals were similar to the overall study population [32 days (IQR 15 days) vs. 46 days (IQR 24 days)]. In the subgroup of patients with cirrhosis, hypertrophy after PVE was more pronounced than after TARE, but the difference was not statistically significant [18.2% (IQR 16.7%) vs. 7.4% (IQR 23.8%); *p* = 0.212]. Time intervals between PVE and postinterventional CT/MRI were shorter than between TARE and postinterventional CT/MRI [34 days (IQR 24 days) vs. 56 days (IQR 10 days)], similar to the overall study population (Fig. 5; Table 3).

**Table 3:** Contralateral liver lobe hypertrophy in patients with cirrhosis and without (after PVE and after TARE)

	Contralateral liver lobe hypertrophy Median (Interquartile range)				
	No cirrhosis		Cirrhosis		
	Hypertrophy	Time interval	Hypertrophy	Time interval	
PVE	25.9% (29.8%)	32 days (15 days)	18.2% (16.7%)	34 days (24 days)	*p* = 0.203
TARE	8.6% (12.8%)	46 days (24 days)	7.4% (23.8%)	56 days (10 days)	*p* = 0.928
	*p* = 0.002		*p* = 0.212		

After PVE, hypertrophy in patients without cirrhosis was more pronounced than in patients with cirrhosis [25.9% (IQR 29.8%) vs. 18.2% (IQR 16.7%); *p* = 0.203]. After TARE, hypertrophy was similar in patients with cirrhosis and without [7.4% (IQR 23.8%) vs. 8.6% (IQR 12.8%); *p* = 0.928] (Fig. 5; Table 3).

### Interrater reliability

For the evaluation of cirrhosis, both radiologists assigned identical scores in 87.7% (64/73) and different scores in 12.3% (9/73). The resulting Cohens kappa was κ = 0.741, indicating substantial interrater agreement.

## Discussion

Our study shows that PVE leads to significantly more pronounced hypertrophy of the FLR in a shorter time interval than TARE, particularly in patients without cirrhosis. These results are in keeping with recent studies, e.g., with a study by Garlipp et al. [6] and with studies by Teo et al. [5, 7]. A possible reason is the large contribution of portal blood flow on total hepatic perfusion and the resulting high impact of occluded portal veins.

In the subgroup of patients without cirrhosis, hypertrophy after PVE was also significantly more pronounced than after TARE, whereas in patients with cirrhosis, there was a similar trend, but this was not statistically significant. The most likely cause for the latter are the relatively low case numbers in these subgroups of the study, as the median for hypertrophy after PVE was still more than twice the median for hypertrophy after TARE.

After PVE, hypertrophy was more pronounced in patients without cirrhosis than in patients with cirrhosis, although the difference was not statistically significant. Indirectly, this result is in keeping with a study by Madoff et al. which showed that sufficient hypertrophy seems to take longer in patients with cirrhosis than in patients without cirrhosis [13]. In contrast, hypertrophy after TARE was similar in patients with cirrhosis and without. A possible explanation is the decreased portal blood flow caused by the cirrhosis and the compensatory higher proportion of hepatic arterial blood flow in regard to total hepatic perfusion [8], resulting in a lower impact of occluded portal veins in patients with cirrhosis. In TARE, where hepatic arteries are occluded and not portal veins, cirrhosis seems to be a minor factor for hypertrophy. This result is in keeping with a recent review by Birgin et al., which showed no significant difference of kinetic growth rate after TARE in patients with and without cirrhosis [17].

In summary, in our study, there was similar hypertrophy of the contralateral liver lobe in patients with and without cirrhosis after TARE, compared to PVE, where hypertrophy in patients without cirrhosis was more pronounced. Thus, cirrhosis seems to be less of a limiting factor for hypertrophy after TARE. Nevertheless, in the complete subgroup of patients with cirrhosis, hypertrophy after PVE was more pronounced than after TARE. Since TARE offers the added potential benefit of local tumor control and downsizing, further prospective studies are necessary to evaluate if TARE could be an alternative to PVE in patients with cirrhosis.

One of the main reasons for the implementation of this study was the known high variability in hypertrophy and the evaluation of possible influencing factors, which are not yet well studied. We showed that cirrhosis is one relevant factor, but there is still a high variability in hypertrophy in each examined subgroup of this study. Hypertrophy ranges from slight increase up to over 90% relative increase in volume. Furthermore, some patients showed decreased volume of the FLR/contralateral liver lobe after PVE/TARE. In the most pronounced case, the FLR shrunk by about 30% after PVE. This patient had cholestasis and therefore likely liver edema in the CT examination before PVE, then received percutaneous transhepatic biliary drainage, and (without repeat CT/MRI examination) PVE was performed. On the follow-up CT scan after PVE, no cholestasis was present, which probably led to the marked decrease in liver volume. In some patients with cirrhosis, we encountered less pronounced shrinking of the contralateral liver lobe after PVE and after TARE. A possible explanation are on average longer time intervals between intervention and follow-up CT scan, which could have led to progression of cirrhosis. The majority of these cases also showed shrinking of the contralateral liver lobe below 10%, which could partially also be due to inaccuracies on volumetry.

Cirrhosis seems to be one relevant influencing factor, but this study indicates that hypertrophy induction is complex and likely dependent on a multitude of factors. Several studies showed that the combination of portal venous embolization and transarterial chemoembolization, sequential or at the same time, can lead to resectability also in patients with large unilobar tumor burden [28]. Another advantage of this method seems to be longer overall and recurrence-free survival in patients with HCC [29]. To evaluate additional aspects, e.g., anatomy of portal veins or choice of embolization agents, further studies will be necessary.

The evaluation of cirrhosis in CT/MRI is a limitation of this study. Liver biopsy, which is the gold standard for diagnosing cirrhosis [30], was not routinely performed in our patient group. However, by using the above-mentioned criteria, we were able to reach substantial interrater agreement for evaluating cirrhosis in CT/MRI. Intraobserver reliability for evaluation of cirrhosis was not performed, which is another limitation of this study. The time interval between intervention and postinterventional CT/MRI after TARE was significantly longer than after PVE. Post-interventional imaging after PVE was performed prior to surgery (median 33 days), whereas imaging after TARE followed routine protocols (median 52.5 days). This renders sufficient matching unfeasible. To evaluate the time course of hypertrophy after TARE and PVE, further studies are necessary. Further limitations are the retrospective study design and the relatively low case numbers.

In conclusion, TARE induces less pronounced hypertrophy of the FLR compared to PVE. Cirrhosis seems to be less of a limiting factor for hypertrophy after TARE, compared to PVE.

## Abbreviations

FLR: Future liver remnant
IQR: Interquartile range
PVE: Portal vein embolization
TARE: Transarterial radioembolization
